# Wearable technologies for health research: Opportunities, limitations, and practical and conceptual considerations

**DOI:** 10.1016/j.bbi.2023.08.008

**Published:** 2023-08-07

**Authors:** Lydia G. Roos, George M. Slavich

**Affiliations:** Department of Psychiatry and Biobehavioral Sciences, University of California Los Angeles, Los Angeles, CA, USA

**Keywords:** Wearables, Wearable technology, Health technology, Measurement, Digital biomarkers

## Abstract

One of the most notable limitations of laboratory-based health research is its inability to continuously monitor health-relevant physiological processes as individuals go about their daily lives. As a result, we have generated large amounts of data with unknown generalizability to real-world situations and also created a schism between where data are collected (i.e., in the lab) and where we need to intervene to prevent disease (i.e., in the field). Devices using noninvasive wearable technology are changing all of this, however, with their ability to provide high-frequency assessments of peoples’ ever-changing physiological states in daily life in a manner that is relatively noninvasive, affordable, and scalable. Here, we discuss critical points that every researcher should keep in mind when using these wearables in research, spanning device and metric decisions, hardware and software selection, and data quality and sampling rate issues, using research on stress and health as an example throughout. We also address usability and participant acceptability issues, and how wearable “digital biomarker” and behavioral data can be integrated to enhance basic science and intervention studies. Finally, we summarize 10 key questions that should be addressed to make every wearable study as strong as possible. Collectively, keeping these points in mind can improve our ability to study the psychobiology of human health, and to intervene, precisely where it matters most: in peoples’ daily lives.

One of the greatest challenges of health research is the relatively high cost and burden of assessing biological processes repeatedly, especially in naturalistic settings. Although less accurate than gold-standard systems such as Biopac ECG that are often preferred in laboratories, wearable technologies—or *wearables* for short—allow for the long-term, scalable, high-frequency quantification of individuals’ ever-changing physiological states, as well as the unobtrusive monitoring of health behaviors in participants’ natural environments (Huhn et al., 2022). In doing so, wearables have increased opportunities for researchers to track and intervene on physiological and behavioral mechanisms central to the field of psychoneuroimmunology (PNI). They have also opened the door to ecological momentary assessment (EMA) of physiological processes (Zapata-Lamana et al., 2020), providing a promising avenue for advancing PNI research.

As wearables have increased significantly in their availability, utility, and accuracy in recent years (Jia et al., 2018; Zhang et al., 2020), so, too, has their use among both among consumers and researchers. Indeed, about 30% of US adults regularly used wearables in 2020—a number that continues to increase exponentially with an expected growth of 24.7% annually through 2026 (Chandrasekaran et al., 2020). In addition, a recent scoping review found that the number of published studies that included noninvasive, consumer-grade wearables increased by 400% from 2016 to 2020 (Huhn et al., 2022). Such work is being enabled in part by an increasing willingness of academic and industry partners to collaborate, which can be mutually beneficial: whereas investigators gain access to the best products and technological support available, companies get higher-quality, academically anchored validation studies that help test the devices and establish their clinical utility.

Wearables are useful in mental and physical health research for many purposes, and one particularly notable purpose is to measure physiological and behavioral processes related to stress. Chronic stress is one of the strongest determinants of lifespan health (Slavich, 2016) and is considered a major modifiable risk factor for noncommunicable diseases associated with poor cardiovascular, metabolic, and immune system health (Fricchione, 2018; Poplawski et al., 2020). Chronic stress is also strongly associated with anxiety and depression (Cohen et al., 2015; McLoughlin et al., 2021; Slavich and Irwin, 2014), making it highly relevant across research topics related to mental and physical health. Stress tracking features in wearables have gained in popularity in recent years, partly due to a greater focus on mental well-being during the COVID-19 pandemic. And although few of these stress-tracking features have been validated in third-party studies as of yet, the use of metrics obtained from wearables to complement more subjective indicators of mental health, which can in turn be used to develop valid machine learning algorithms, has great potential for advancing health research. As such, we use examples related to stress throughout this paper to highlight the utility of wearables in this context.

Despite the growing popularity of using wearables in studies and their potential for advancing PNI and health research more broadly, there are few guidelines for using wearable technology in research studies and little shared understanding of the key considerations for choosing a wearable for research use. In addition, there are several limitations of wearables that must be taken into account when choosing *whether* to use wearables in a study and *which product* is the best match for the goals of the study. For example, validity differs across devices and metrics, depending on the hardware, software (including algorithms), and data quality choices, such as sampling rate. In addition, the ways in which the participant and the researcher will interact with the data must also be considered in light of the study goals and the need for a positive participant experience. The first aim of this article, therefore, is to provide researchers with an overview of typical physiological metrics that can be derived from wearables (i.e., “digital biomarkers”) as well as behavioral indices that can be used in PNI research. The second goal is to describe the potential uses and benefits of wearable technology in PNI research and health research more broadly, as well as the limitations and potential pitfalls, including key considerations for choosing a wearable device for research use.

## 1. Metrics obtained from wearables

As the number of sensor types and features offered by wearables continues to grow, so does the amount of data researchers can collect unobtrusively and relatively continuously. Of note, several other types of portable devices and tools exist that can be integrated into daily life and used for research. These include smartphone and web apps for clinical monitoring (e.g., Sverdlov et al., 2021); facial and vocal recordings for assessing stress and clinical disease processes, including the use of artificial intelligence and machine learning in feature detection (e.g., Chandrabhatla et al., 2022; Kappen et al., 2023; Lim et al., 2022); and pollution-monitoring devices for assessing particulate matter (PM) 2.5 exposure (e.g., Gao et al., 2022; Jiang et al., 2018). In this article, we focus on the most commonly used types of wearable devices that are available to both consumers and researchers. In doing so, we describe the most prevalent technologies commonly used among consumers and health-focused researchers (e.g., smartwatches/bands, rings), as well as examples of health-relevant metrics that can be obtained from them. A summary of these metrics is provided in Table 1.

### 1.1. Accelerometers and gyroscopes

Accelerometers and gyroscopes were among the first sensors used in both research- and consumer-grade wearable technology to measure motion data. Accelerometers detect acceleration to provide data on whether—and how fast—an individual is moving, and gyroscopes measure angular velocity to determine the orientation of the wearable in space. These sensors remain widely used today, as their combination allows for the assessment of whether physical activity is occurring, what type of activity is happening, and whether a person is upright or lying down. Information obtained from the accelerometer and gyroscope also aid in the removal of motion artifacts that may be present in data derived from other sensors.

### 1.2. Photoplethysmography

Photoplethysmography (PPG) uses optical sensors that index changes in blood volume underneath the skin’s surface by emitting a light-emitting diode (LED) into the skin and then measuring the amount of light absorbed using a photodetector. These LEDs are often green, but may be red, infrared, or white spectral light (see below for considerations related to LED color). Several biomarkers can be estimated using PPG signals, including heart rate and heart rate variability (HRV), blood pressure, respiration rate, and blood oxygen saturation (SpO_2_). In addition to examining physiological processes, wearables can be used to investigate health behaviors that act as both precursors to—and consequences of—stress responses and changes in mental and physical health. For example, physical activity and sleep behaviors can be estimated using a combination of accelerometer, gyroscope, and PPG sensing.

### 1.3. Electrodermal activity

Electrodermal activity (EDA) sensors measure small changes in the electrical conductance of the skin due to variation in sweat production, which is then used to estimate sympathetic nervous system activity that is predictive of health (Dedoncker et al., 2022; Slavich et al., 2023). Newer to the consumer-grade wearable market, EDA has yet to be widely validated in wrist-worn wearables. Some wearable rings also measure EDA, which boast more similarity to gold-standard Biopac EDA measurement—typically conducted using the fingertips or palm where there are more sweat glands—but also have yet to be validated to the extent that we can be relatively certain of their accuracy compared to research-grade EDA sensing devices.

### 1.4. Thermometers

A more recent development in wearables is built-in thermometers for temperature tracking. Although they are not a replacement for core temperature thermometers, they can be used for monitoring changes in peripheral skin temperature (Smarr et al., 2020). Some wearables now use peripheral temperature to gauge when a user may be sick (assessed by significant increases in temperature above the user’s typical range) and to estimate processes related to reproductive health, such as ovulation. In the future, temperature monitoring may also be useful for detecting stress (Herborn et al., 2015), as acute stress is known to trigger peripheral vasoconstriction and cause rapid, transient drops in skin temperature.

### 1.5. Global positioning systems

Outside of the direct measurement of physiological processes, wearables with built-in global positioning systems (GPS) or with the ability to connect to a paired phone’s GPS can measure changes in location. For example, location variance has been associated with depression (Moshe et al., 2021), partly because lack of variability in locations traveled may serve as an objective indicator of social isolation. In addition, researchers examining an individual’s environment as part of a study can use GPS to understand, for example, whether participants spend most of their time in urban or rural areas, socioeconomically advantaged or disadvantaged areas, if they typically reside in a food desert, and potential toxin exposure, all of which are relevant for monitoring health risks.

### 1.6. Surveys collected via connected apps and externally-linked software

Finally, wearable-connected apps and externally-linked surveys can also request users to enter information on health behaviors on a daily basis to gain self-report data on behaviors that cannot be measured directly, such as alcohol use or time spent working or socializing. Similarly, physical symptoms that are unable to be measured using current technology (e.g., fatigue, pain) and psychological states (e.g., depressed mood) can be obtained using a daily survey in the wearable’s app. Capturing these data alongside physiological metrics allows for EMA and daily diary studies to be conducted with more ease than requesting surveys using a separate platform, and enables the aggregation of data over time for a snapshot of an individual’s day-to-day physiology, functioning, behavior, and experiences more accurately than laboratory-based studies that rely on participant recall. Often, researchers collect data on these topics using surveys administered on a separate software or app. However, some wearable apps provide the option for daily “journal” entries that collect data on health behaviors, perceived stress, and mood (e.g., WHOOP), whereas others enable researchers to customize the variables on which participants can report (e.g., Biostrap).

## 2. Uses and benefits of wearables in research

There are several benefits to using wearables in PNI research in general, and especially in studies of stress and health. At the most basic level, researchers can use wearables to compare basal physiological functioning and behaviors between populations. For example, investigators can examine how resting HR (e.g., computed as the average heart rate when relatively still over the span of a couple of weeks) differs depending on exposure to major stressors over the lifetime, which has been found to predict health (Slavich, 2016), or between a sample of healthy adults and those with posttraumatic stress disorder, depression, or diabetes (Koch et al., 2019; Sadeghi et al., 2022).

Further, because wearables can track intra-individual changes in biomarkers over time, researchers can monitor and investigate both between- and within-person changes minute-to-minute, hour-to-hour, day-to-day, week-to-week, or month-to-month. In doing so, wearables can provide real-time feedback to researchers on chronic disease management and progression, as well as early disease detection (Vijayan et al., 2021). For example, the COVID-19 pandemic saw a new use for wearables in early detection of infectious disease even before users realize they had an illness, often using “donated” wearable data from participants who already owned a wearable to develop algorithms (e.g., Alavi et al., 2022; Mishra et al., 2020). Others have used wearables to help develop machine learning algorithms that can identify the presence of mental health disorders (e.g., depression) and monitor clinical treatment response (Griffiths et al., 2022; Hickey et al., 2021; Lui et al., 2022; Sheikh et al., 2021). Of note, although the uses thus far have shown promise for the ability of wearable devices to detect and monitor diseases (Dunn et al., 2018), the level of accuracy in wearable devices—especially consumer-grade devices—and the variation in algorithms that have been tested thus far do not yet yield sufficient support for using wearables to inform clinical decisions and monitor diseases on their own. Until more research validation is completed, a strong evidence base of safety and effectiveness exists, and data security can be guaranteed, appropriate caution is needed to ensure that patients are not harmed by using wearable devices in health care (Mattison et al., 2022).

Outside of disease detection and monitoring, researchers may, for example, wish to understand how metrics related to stress, psychological well-being, or emotions (e.g., heart rate, HRV, blood pressure, EDA) change in response to an acute natural stressor or an intervention (e.g., Hickey et al., 2021). Conversely, aggregating averages of digital biomarkers over multiple weeks or months enables researchers to estimate shifts in chronic states that are not necessarily related to a clinical disorder.

Importantly, data derived from wearables and their apps enable researchers to observe day-to-day differences within people to better how psychological, behavioral, and physiological factors are interlinked in naturalistic settings over time. For example, investigators can examine how psychological factors captured using measures within the app, like feeling anxious—or health behaviors, like the amount of sleep and quality of sleep—affects physiological indicators of stress and health, like heart rate variability, both over the short- and long-term. Similarly, researchers can investigate how changes in physiological indicators of stress affect participants’ likelihood of engaging in certain health behaviors, as well as self-reported affect and cognitions. For instance, researchers may ask questions like: How do fluctuations in HRV and resting heart rate affect the likelihood of drinking alcohol?

Moreover, a newer use of wearable technology is using digital biomarkers to develop machine learning algorithms that can identify when a participant experiences a distinct physiological state, such as when a stress response is occurring, to understand how psychosocial and physiological processes unfold in the real world. For example, several research groups have created algorithms to detect acute stress responses in laboratory and naturalistic settings (e.g., Anusha et al., 2018; Arza et al., 2019; Chalmers et al., 2021; Nath et al., 2022; Sandulescu et al., 2015). Using such algorithms, researchers can test hypotheses related to stress processes in real time to understand how situational and individual factors affect stress responses and rates of recovery. Further, by using these data to develop machine learning algorithms, we have the potential to conduct real-time stress monitoring and guide just-in-time interventions that can enhance adaptive recovery.

Finally, wearables enable researchers to collect physiological and behavioral data remotely. Remote data collection continues to become more common as researchers develop new processes to improve participant recruitment, reduce the likelihood of “white coat phenomenon” affecting data, and expand participant reach. Remote data collection became even more popular when the COVID-19 pandemic required many laboratories to shut down in-person recruitment. In fact, a survey of 245 researchers found that the proportion of interactions with participants conducted remotely changed from 9% in January 2020 to 57% in May 2020 (McDermott and Newman, 2021). For many studies examining physiological processes, wearables made this shift to remote research possible.

## 3. Limitations and pitfalls of using wearables in research

Wearable devices have many potential uses and possible benefits for research. However, several limitations also exist, of which researchers should be aware. Despite physiological and behavioral indices derived from wearable data often being called “objective”, for example, the reality is that there are many relatively subjective decisions made throughout the process of wearable technology and algorithm development that affect how data are collected, managed, and analyzed prior to the researcher receiving the data that influence the final numbers and ways in which the data can be accurately interpreted. We describe several of these points below.

### 3.1. Measurement-related limitations

First, wearable companies must balance data quality with other needs, such as battery life, item size, and other hardware and software features. Because most consumers are primarily concerned with a device’s features, battery life, and screen quality, data quality is not often the top priority. In addition, companies are pressured to produce new features before competitors, many times prior to the technology being externally validated and sometimes even before they have been internally validated. This validation work is perhaps even more important in cases where the hardware differs significantly from what is used in research. For example, multiple wrist-worn wearables now measure EDA—despite research suggesting that EDA derived from the wrist is less valid than EDA derived from the fingertips or palms (Hossain et al., 2022; van der Mee et al., 2021)—with no published studies validating their specific product’s EDA feature.

Second, few consumer-focused companies provide researchers access to raw, unfiltered data. Researchers must thus use computations derived from companies’ proprietary algorithms, the details of which are not always made available to the public. For example, some wearable products track HRV overnight. However, whereas some average all HRV data from the entire night, others use different methods for producing the final HRV reading for the night. For example, they may calculate HRV by compiling HRV data from multiple sleep stages or calculate the average from the last 5 min of every “deep sleep” state, which means the interpretation of the nightly HRV reading is dependent upon the accuracy of the sleep staging algorithm. These differences in algorithms are perhaps not as important in a single research study—for example, when assessing change within-person over time—but the variability in methods for obtaining HRV during sleep become very important when attempting to aggregate or compare results across studies. Further, it is important that researchers are aware of the ways in which the technology and algorithms are employed to ensure that the data produced by the wearables fit the needs of the study.

An issue central to diversity and racial equity, and one that is common to many wearables, is the use of optical sensors that are less accurate in darker skin, and in those with freckles and tattoos (Koerber et al., 2022). Most wearables use green LED lights because they reduce signal distortion and “noise” in the data, and typically cost less; however, green light is easily absorbed by melanin the skin. In darker skin tones, therefore, it may not penetrate the skin enough to pick up an adequate signal. Some wearables have moved to adding red and infrared LED lights as well, which are not as easily absorbed and thus allow for more accurate PPG signaling (Nelson et al., 2020). Nonetheless, even red LED lights are not as accurate in darker skin—an issue that garnered more attention since the COVID-19 pandemic shone a spotlight on healthcare inequities related to pulse oximeters in medical settings, which use red LED lights (Cabanas et al., 2022). In fact, prior research has found that these lights can be up to 15% less accurate in persons with darker skin (Bent et al., 2020).

To increase PPG accuracy in darker skin tones, some devices such as Fitbit and Apple Watch, have a feature that boosts the intensity of the green light when the device is having trouble acquiring an adequate PPG signal. A small portion of devices have started using white spectral light in addition to red and green LEDs for a multi-wavelength solution that can provide more accurate PPG signals across a wide range of skin tones.

Although some companies have begun to develop solutions for ensuring wearable accuracy in varied skin colors, much less attention is granted to diversity in body size. Excess body fat increases skin thickness and alters blood flow and oxygen saturation, which affects optical properties of the skin and the extent to which light can travel through the skin and may affect the amplitude and accuracy of PPG signaling (Fine et al., 2021). For example, a Monte Carlo simulation predicted a 40% loss of PPG signal amplitude in obese individuals due to increased skin thickness on the wrist with a simulated radial artery increase from 2.5 to 3.5 mm (Hirt et al., 2019). However, *trans*-epidermal water loss is also common in people with higher body mass indexes, which may increase PPG signal intensity (Rodrigues et al., 2017). However, research on the topic of body fat and wearable accuracy in real participants is limited. Research that has been conducted on body fat and PPG accuracy has used samples from particular populations (e.g., only women with diabetes) and focused on wrist-worn wearables. Therefore, whether differences in accuracy exist across populations and at other body sites (e.g., the finger) is unknown (Fine et al., 2021).

Further, excess body fat may contribute to differences in electrodermal activity. For example, a study that compared obese and non-obese *cis*-men and women found that skin conductance responses differed significantly depending on obesity status across sex (Aldosky, 2019), possibly due to subcutaneous fat eliciting greater sweat production (Shipman and Millington, 2011; Yosipovitch et al., 2007). However, more studies are needed to understand this association more fully. In light of these potential sources of inaccuracy across diverse populations, researchers must seek validation data on wearables they are considering using in their studies and ensure that the wearable is validated in the population of interest, or at the very least uses technology that is likely to provide more accurate PPG and electrical signaling in the target population.

Researchers must also be aware of—and account for—the use of certain medications that may disrupt inferences that can be made with the data. For example, common medications for attention-deficit/hyperactivity disorder (ADHD) increase sympathetic nervous system activity. If a participant only uses the medication on some days, it may make it more difficult for researchers to detect differences in physiological processes that are due to stress responses or behaviors unless medication use is accounted for in data analysis. These medications can also create ceiling effects, such that if the nervous system is highly activated when the participant uses stimulants, such as medications commonly prescribed for ADHD, it may be more difficult to detect increases due to stress. Conversely, some medications (e.g., blood pressure-lowering medications, anti-depressants) that reduce sympathetic nervous system activity may dampen stress responses.

Finally, wearable devices vary in their accuracy. For example, research suggests that some wearables may underestimate heart rate and inaccurately estimate activity, such as steps (e.g., Benedetto et al., 2018; Tedesco et al., 2019). Different sensors on wearable devices and the fact that companies typically use different timing and algorithms to estimate the same metric (e.g., resting heart rate) largely prohibits direct comparison of information across wearable devices, especially when examining between-person effects. As such, in addition to verifying the validity of the metric of interest with the device that will be used by participants (discussed below, under “Key Considerations When Choosing a Wearable for Research Use”), it behooves researchers to use a single type of device across all participants when they will be comparing effects between participants and restrict participants to a single device type over the course of the study if they will be examining within-person effects over time.

### 3.2. Stress-related limitations

Several consumer-grade wearables claim to monitor psychological stress, and many studies have differentiated experimental stress conditions in research participants using physiological signals (e.g., Anusha et al., 2018; Arza et al., 2019; Chalmers et al., 2021; Herborn et al., 2015; Mozos et al., 2017; Nath et al., 2022; Sandulescu et al., 2015). However, little external validity has been shown, and wearables are not yet at the point of being able to reliably detect when a person is having a stress response. Nonetheless, researchers are honing in on procedures for bridging stress detection in laboratory-based experiments with digital phenotyping in naturalistic environments (Egger et al., 2020). With continued research, we may be able to identify stress responses with relative accuracy. This section applies to researchers who are attempting to develop and validate processes and algorithms for identifying stress responses, as well as researchers who expect to use these processes in their own once validated.

Regardless of the device’s accuracy for estimating physiological processes, it is important to note that not all physiological data limitations and potential sources of error derive from the device itself; an additional hurdle arises with the ability to interpret the data accurately. Researchers using wearables to assess stress responses must ask questions such as: *Is the device detecting a psychological stress response (e.g., as opposed to a stress response during exercise)?* Oftentimes, the focal physiological signals themselves do not provide this information, and it is necessary to use additional metrics provided via the wearable. For example, broadly speaking, we can use an accelerometer and gyroscope—features common to most wearables—to determine when a person is moving and, therefore, when a stress response is occurring in response to exercise (i.e., a physiological stressor). Many wearables now also have the capability to track temperature changes, enabling researchers to evaluate if heightened autonomic activity (e.g., higher heart rate, lower HRV, more EDA responses) may be due to illness.

If we can be reasonably certain that the stress response is not a result of experiencing a physiological stressor, we must then consider the type of psychological stress response. Observational stress research is limited in differentiating types of psychological stress responses. Stressors, and the responses humans have, occur on a spectrum of affectively positive (exciting) and negative (dangerous); challenging, with positive outcomes, and threatening, with potentially harmful outcomes; adaptive (necessary, acute) and maladaptive (unnecessary, chronic). Although distinct in their psychological and behavioral processes and outcomes, as well as chronic health implications, acute physiological responses observed via current wearable technology may appear indistinguishable. Researchers must therefore establish processes for asking questions such as: *Is it a positive or negative stress response? Is the stress response acute, with a relatively fast return to baseline (i.e., recovery)?* Because of these limitations in terms of what we can interpret from physiological data alone, it is preferable to use behavioral and survey data captured within an app alongside the physiological data for a fuller picture.

As mentioned above, some apps connected to wearables include the option for a daily journal entry or survey that has the capacity to ask questions about perceived stress, anxiety, depressive mood, and other cognitive and affective responses. These surveys can also capture additional behavioral data like potential coping behaviors (e.g., alcohol use), which can provide insight into the effects of the stress response on physiology and potential longer-term health and well-being via behavior, in addition to health behaviors discerned by the wearable automatically, such as sleep quality. In many cases, apps that accompany the wearable do not have the capability or flexibility to add customized survey items; in those cases, many researchers opt for linking surveys using separate software or apps.

If the goal is to detect and understand stress responses in the moment, wearables and their associated apps can prompt participants to answer a few short questions following a detected stress response to confirm the validity of the physiological signal, ascertain the type of stressor, and gather information about emotions and cognitions. Importantly, the prompt should be generated shortly after the stress response (e.g., within five minutes) to get the most accurate data (Weber et al., 2022). It is also helpful if the user has the ability to log exposure to stressors or stress response via the wearable or its related app. For example, WHOOP can ask users to log “High Stress Work” as an activity with a start and end time. However, this is not a common feature among wearables, and it may behoove researchers to examine whether a particular wearable offers this feature or a potential workaround (e.g., logging an exercise as “Other”) that will enable researchers to collect these data if it would be beneficial for the study.

Finally, it is important to keep in mind that stress responses are highly variable, and that not all stress responses can be estimated using the same parameters. Prior research has found that variability in stress response patterns—including the extent to which the autonomic nervous system and hypothalamic–pituitary–adrenal (HPA) axis is activated, which then affect immune system response and recovery—is attributable in part to situational characteristics of the stressor as well as individual appraisal of those characteristics (Schlotz, 2013). For example, some research has found that whereas stressors requiring cognitive and physical effort often elicit autonomic responses, stressors involving social evaluative threat frequently trigger increased HPA-axis activity (Dickerson and Kemeny, 2004). As several sweat-based cortisol monitoring prototypes are being developed, it is also becoming apparent that noninvasive, wearable-based cortisol sensing may also soon be possible (Samson and Koh, 2020; Torrente-Rodríguez et al., 2020; Wang et al., 2022). For now, though, researchers might consider combining wearable data with salivary cortisol to better understand and contextualize stress responses.

Indeed, a study examining within-person differences in stress system reactivity across four physical, cognitive, and social-evaluative tasks found evidence for distinct stress response patterns depending on the type of stressor (Skoluda et al., 2015). Heightened autonomic activity was found across all stressors compared to rest, although the response intensity differed; for example, heart rate increased the most in response to the exercise task (Ergometer), followed by responses to the social evaluative (Trier Social Stress Test) and cognitive (Stroop) tasks, which were similar to one another, and no significant response to the temperature stressor (Cold Pressor Task). However, HPA axis activity measured via cortisol showed distinctly higher responses to social evaluation than to the exercise and temperature stressors, and no significant response to the cognitive stressor (Skoluda et al., 2015).

Further, studies have found differences in the extent to which individuals exhibit autonomic versus cortisol responses to stress. For example, heart rate and cortisol responses to stressors are not always correlated (e.g., Bönke et al., 2019), and studies have found that although a stressor may elicit similar autonomic responses across participants, only a portion of the participants (e.g., 60%) experience a cortisol response (Schwabe et al., 2008). In fact, a great deal of variability in cortisol stress responding exists, with a significant proportion of individuals being classified as “cortisol non-responders” (Miller et al., 2013). We see these differences in cortisol reactivity even when exposed to the Trier Social Stress Test, widely known as the most reliable way to induce cortisol responses in study participants (Allen et al., 2017). Although it is often not possible to assess all potential stress responses, it is important to remain cognizant of the potential for differences across physiological stress systems and refrain from assuming that responses in one system reflect activation of all stress systems and processes.

## 4. Key considerations when choosing a wearable for research use

Wearable devices differ in their features, accuracy, data types, sampling rate, user experience, pricing model, and cost. Below, we provide 10 key considerations to keep in mind when choosing a wearable device to measure digital biomarkers. (Also shown in Table 2.) They are:

1. The first question to consider is: Is the *metric of interest* validated? For example, you may ask the question: Is HRV a reliable indicator of psychological stress? This may require conducting literature reviews and weighing the evidence of various metrics in the particular context and population of interest. Although outside of the scope of this paper, in-depth reviews on these topics would be very helpful, and we recommend conducting future studies and reviews that focus on verifying the validity of wearable-derived metrics for various constructs.
2. Second, is the *device* validated for *that metric*? For example, if you are interested in working with the Oura ring, has HRV assessed via the Oura ring been validated against the gold standard (e.g., ECG)? When appropriate, (e.g., for SpO_2_ measurement,) you may even ask whether the device’s metric has been FDA-approved. If the device has not been validated for that metric, are features of the device at least similar to other products that have been validated? For example, you may consider that EDA devices used in laboratory studies use finger- and palm-based monitoring systems because they are better able to detect sweat than on the wrist. Logically, then, it would follow that finger-worn wearables such as rings may provide more reliable data devices than wrist-worn wearables.
3. Third, how *equitable* is the device, especially for your metric and population of interest? Has the device been validated across diverse samples? Has your metric of interest been validated in a sample that is representative of your target population using this device? If data are scarce, consider how the accuracy of wearables with similar technology have fared in those populations. In addition, be sure to consider the accuracy of the technology (e.g., the lights used for PPG) as well as any algorithms the company uses to improve accuracy.
4. Fourth, does the metric *sampling interval match* what is needed for the research question? For example, although Apple Watch provides a relatively accurate heart rate, its heart rate and HRV sampling rate differs depending on the activity; it samples heart rate every 5 or 6 s and its accuracy outperforms many other wearables in terms of accurate heart rate measurement during exercise, especially for activities like resistance training (Støve and Hansen, 2022). As such, the Apple Watch may be a fitting option for assessing heart rate during exercise or other activities that can be assessed with the exercise function. However, it would be a poor choice for a study aimed at estimating HRV during sleep, as it only measures HRV once every hour. As such, it may collect the data during relatively random sleep stages which result in wildly different HRV scores and impractical information when averaged for a nightly HRV score. Conversely, the Oura ring assesses HRV every five minutes throughout the night. A similar consideration is the extent to which companies are willing to publicize information on aspects of their wearables, such as about details and algorithms they deem proprietary, and especially if the information has the potential to be harmful to sales (e.g., study results that do not support the wearable’s validity).
5. Fifth, how will participants interact with data? Consider questions like: Do you want participants to be able to see their data, or might seeing their data change their behavior in ways that could alter the results of the study? If you do not wish participants to see their data throughout the study, are you able to collect and store the data outside of an app with which the participant must interact? If you would like to use a separate app to collect the data, does the company have an open API that will allow you to sync the data with the other app? Does the app have the capability to request survey responses—for example, using modified journal entries—from participants on topics that are central to the researcher’s aims (e.g., about affective responses and cognitions throughout the day, behaviors engaged in)?
6. Sixth, *what* data will you receive and *how* will you receive the data, particularly if you are using the company’s app? What indices will you receive? Do you need raw, unfiltered data (e.g., every HRV data point throughout each night)? If so, will the company provide that to you, and do you have the analytic capabilities to work with unfiltered data? Or will you receive summaries (e.g., average HRV score for each night)? If receiving summarized data, how are the summary scores calculated and are they adequate for answering your research question? Similarly, missing data and identifying non-wear time as well as erroneous data points will be important considerations, along with whether the company will clean data or if you, the researcher, will be responsible for data cleaning. As a general rule, whenever possible, authors’ rationale for making decisions, and the code and/or tools used to calculate scores, should be included in the supplementary materials of relevant publications to enable reproducibility and help promote open science.
7. Seventh, what is the *participant’s experience* of the device and app? Is the device comfortable? How long is the battery life? Can it be charged while worn? Is the device waterproof or at least water resistant? Is the platform intuitive, engaging, and easy to use? Are the built-in surveys short and easy to answer? These considerations are essential for understanding participant burden and can affect the likelihood that your participants will wear the device often enough for data to be useful and provide additional survey responses via the app. Along with the participant’s overall experience of the device and app, digital health literacy and connectivity are important considerations, particularly if participants must own a smartphone capable of syncing wearable device data to ensure equitable participation opportunity and ease of use. When needed, researchers should be prepared to provide smartphones (e.g., on loan) with data plans included to participants and provide ample education about how to use the wearable and smartphone devices.
8. Eighth, does the wearable and app protect confidentiality and privacy to the extent possible? Investigators must prioritize participant confidentiality and privacy as with any product or tool used in research. In the United States, HIPAA protection does not extend to health- and location-related data collected using wearable technology and connected devices, and the terms and conditions of use by private companies are often not transparent or protective of consumers. Researchers must thus take steps to ensure that data derived from wearables and any accompanying apps are secure and HIPAA-compliant by identifying how—and to what extent—companies safeguard their data. Relatedly, researchers should ensure that data collection and storage procedures comply with all applicable laws and regulations. They must also be aware of potential privacy concerns, given the vast information about health, behaviors, and potentially location, obtained by wearables, particularly if connected with sensitive personal information from survey data. Wearable devices—as with most other advanced technologies—can be hacked, and personal information stolen. (For in-depth reviews of privacy and security concerns, see Datta et al., 2018; Kapoor et al., 2020.) Finally, researchers should ask to whom the data belong: the participant, the researcher, or the wearable company? Might the company share data with third parties or use it internally for any reason?
9. Ninth, do you have the research staff needed to train all participants in how to use the device and to provide technical support when needed? Research staff are also needed to remotely monitor data quality to ensure that participants are both wearing and syncing their devices, and that the wearable data are successfully being uploaded to the cloud, if applicable. These participant compliance checks are critical, especially when studying older or chronically ill participants, or those with many demands or limited technological knowledge. Even with adequate support, it is not uncommon for participants to have missing data due to device failures, problems with syncing, and other technical issues. Missing data should therefore be expected when using these tools, and researchers should be prepared to use appropriate statistical methods for handling missing data.
10. Finally, the tenth consideration is the pricing model and overall cost to the researcher. How much does the wearable cost and does the pricing model (e.g., one-time price vs. monthly) match your needs and budget? Many wearable device companies have a one-time price that vary widely (e.g., $200-$700), although some companies have moved toward an alternative pricing model wherein the researcher leases the wearable and accompanying app on a monthly basis (e.g., $30/month). Further, some companies require both an upfront and monthly payment. Depending on the length of the study, differences in price over time may become a relevant factor in deciding on a wearable device. Although the monthly leasing model may be the best option for a single study in which participants wear the device for one month, purchasing the device outright may be appropriate for researchers who will need the devices for long periods of time or who expect to use the same devices for multiple studies. In addition, some companies that lease on a monthly basis require agreement to a minimum contract (e.g., a year). Central to the idea of cost is also the ability of the device and platform to “deploy and forget”. That is, if you have rolling participation, can you use the same device for multiple participants in succession? If so, how many sampling days are required before you have reliable data and can then transfer the device to a new participant?

Some information frequently needed by researchers can be found on company websites (e.g., cost). As wearables have become more commonly used in academic research, however, vendors have begun providing information about using their devices and apps in academically focused webinars and publicly available documents. Once researchers have narrowed down their options, the next step is to contact a company representative—for example, a sales representative or academic partnerships liaison—to describe the goals of the project or lab, ask any remaining questions, and, if needed, set up processes unique to the study, such as incorporating study-specific measures or items into the devices’ app.

## 5. Conclusion

In conclusion, one of the greatest strengths of PNI research—namely, its focus on the high-quality characterization of health-relevant biological processes—has paradoxically also been one of its greatest limitations, since the technologies needed to obtain high-quality physiological data historically required in-person study visits. As a result, PNI researchers have not been able to continuously monitor health-relevant processes as people go about their daily lives and experience positive and negative life events. Wearables have the potential to change that. If combined with remote psychological and blood microsampling techniques, for example, wearables can provide PNI researchers with extremely rich, multi-level data that elucidate how psychological, physiological, immunological processes change in response to daily naturalistic experiences. Such continuous monitoring can also supply the empirical data needed to guide just-in-time adaptive interventions aimed at intervening before pre-clinical and clinical disease processes take hold or to aid treatment. If calls for proper data collection and processing standards are heeded (Nelson et al., 2020), wearables have the potential to revolutionize what is possible in PNI and health research.

## Figures and Tables

**Table 1:** Summary of commonly used metrics obtained from wearables.

Metric	Technology
*Physiological*	

Heart rate – resting	PPG
Heart rate – reactive (e.g., during a logged exercise)	PPG
Heart rate variability (typically overnight)	PPG
Blood pressure	PPG
Respiration rate	PPG
Blood oxygen saturation (SpO_2_)	PPG
ECG	Electrical heart sensor
Skin temperature	Temperature sensor
EDA/galvanic skin response/skin conductance	EDA

*Behavioral – Physical Activity*	

Step count	Gyroscope, accelerometer
Physical activity types	PPG, gyroscope, accelerometer, GPS
Physical activity duration	PPG, gyroscope, accelerometer
Metabolic equivalent for the task or minutes spent in “heart rate zones”	PPG
Sedentary behavior (e.g., total time spent sitting, sitting events, number of periods of prolonged sitting time)	Gyroscope, accelerometer

*Behavioral – Sleep*	

Total sleep time	PPG, gyroscope, accelerometer
Sleep efficiency	PPG, gyroscope, accelerometer
Sleep onset latency	PPG, gyroscope, accelerometer
Wake after sleep onset	PPG, gyroscope, accelerometer
Sleep stages	PPG, gyroscope, accelerometer
Time in bed	Gyroscope, accelerometer

*Behavioral – Location*	

Location variability	GPS
Distance traveled	GPS
Time spent at home and/or work	GPS

*Note.* PPG = photoplethysmography; ECG = electrocardiogram; EDA = electrodermal activity; GPS = global positioning system.

**Table 2:** 10 key considerations when choosing a wearable for research use.

1. Is the metric of interest validated?
2. Is the device validated for that metric?
3. How equitable is the device, especially for your metric and population of interest?
4. Does the metric sampling interval match what is needed for the research question?
5. How will participants interact with the data?
6. What data will you receive and how?
7. What is the participant’s experience of the device and app?
8. Does the wearable and app protect confidentiality and privacy to the extent possible?
9. Do you have the research staff needed for training participants and providing technical support?
10. Does the cost and pricing model of the wearable match your needs and budget?

## Data Availability

No data were used for the research described in the article.
